# Photobiomodulation in Ophthalmology: A Comprehensive Review of Bench-to-Bedside Research and Clinical Integration

**DOI:** 10.7759/cureus.69651

**Published:** 2024-09-18

**Authors:** Diksha Garg, Sachin Daigavane

**Affiliations:** 1 Ophthalmology, Jawaharlal Nehru Medical College, Datta Meghe Institute of Higher Education and Research, Wardha, IND

**Keywords:** clinical applications, corneal wound healing, low-level laser therapy, ophthalmology, photobiomodulation, retinal diseases

## Abstract

Photobiomodulation (PBM), also known as low-level laser therapy, is an emerging therapeutic modality in ophthalmology, attracting increasing interest for its potential to manage a variety of ocular conditions. PBM employs low-energy light within the red and near-infrared spectrum to penetrate biological tissues, where it interacts with cellular chromophores. This interaction is believed to enhance mitochondrial function, boost adenosine triphosphate (ATP) production, and reduce oxidative stress, leading to improved cellular repair and tissue regeneration. Recent bench research has demonstrated PBM's efficacy in cellular and animal models, showing its ability to modulate inflammatory processes and promote healing in retinal and corneal diseases. For instance, in retinal models, PBM has been observed to reduce apoptosis and support cell survival under stress conditions. Similarly, studies in corneal models suggest that PBM can accelerate wound healing and reduce scarring. Clinical trials further corroborate these findings, revealing that PBM can enhance treatment outcomes in several ocular diseases, including age-related macular degeneration, diabetic retinopathy, and dry eye disease. Patients undergoing PBM have reported improvements in visual acuity, reduced retinal inflammation, and better tear film stability, highlighting its potential as an adjunctive therapy. This review also explores the integration of PBM into clinical practice, discussing current treatment protocols, safety considerations, and the latest advancements in PBM technology. By offering a holistic overview, the review aims to provide clinicians and researchers with valuable insights into PBM’s role in modern ophthalmic care, emphasizing its potential to enhance treatment strategies and improve patient outcomes.

## Introduction and background

Photobiomodulation (PBM), also known as low-level laser therapy, is a therapeutic approach that utilizes light to influence biological processes at the cellular level [1]. This technique involves applying light to target tissues, typically in the red or near-infrared. The primary principle behind PBM is the absorption of photons by cellular chromophores, such as cytochrome c oxidase. This absorption triggers biochemical changes within the cell, including enhanced mitochondrial function, increased adenosine triphosphate (ATP) production, and reduced oxidative stress [2]. These effects collectively promote cellular repair, regeneration, and overall tissue healing. Historically, PBM's development began in the mid-20th century when researchers first observed the effects of low-level laser light on cellular growth and wound healing. Since then, advancements in laser technology and a deeper understanding of its biological mechanisms have expanded PBM’s applications [3]. Modern PBM devices, including diode lasers and light-emitting diodes (LEDs), offer precise control over parameters such as wavelength, intensity, and delivery mode, broadening PBM’s clinical potential [4].

PBM has emerged as a promising modality in ophthalmology due to its potential benefits in managing various ocular conditions. For retinal diseases, PBM has shown promise in reducing inflammation, enhancing retinal cell survival, and improving visual function [5]. In treating corneal and anterior segment disorders, PBM accelerates wound healing, alleviates dry eye symptoms, and potentially minimizes complications following refractive surgeries. The non-invasive nature of PBM, along with its ability to modulate inflammatory responses and expedite tissue repair, positions it as a valuable adjunctive therapy in ophthalmic care. Its integration into ophthalmic practice reflects a shift toward minimally invasive, drug-free treatments that can complement traditional therapeutic approaches [6]. As the field of ophthalmology advances toward personalized and precision medicine, PBM’s ability to target specific cellular pathways and enhance treatment outcomes underscores its relevance and potential in modern ophthalmic care [7].

This review aims to thoroughly examine PBM's role in ophthalmology, encompassing both foundational research and clinical applications. It seeks to consolidate bench-to-bedside research, evaluate the evidence supporting PBM's efficacy and safety, and explore its integration into routine ophthalmic practice. This review will offer valuable insights for clinicians, researchers, and policymakers by synthesizing key findings, technological advancements, and clinical outcomes. The goal is to highlight PBM’s potential to enhance ophthalmic care and to guide future research and clinical applications in this evolving field.

## Review

Methodology 

This manuscript presents a comprehensive review of PBM in ophthalmology. A narrative review format was chosen to allow for flexibility in covering the broad spectrum of research on PBM, ranging from cellular mechanisms to clinical applications, without the constraints of a systematic review. To gather relevant literature, we thoroughly searched databases, including PubMed, Web of Science, and Google Scholar, without restricting the publication date to ensure both historical context and recent developments were captured. The search was performed using a combination of keywords such as “Photobiomodulation,” “Low-level laser therapy,” “Ophthalmology,” “Retinal diseases,” “Corneal healing,” “Mitochondrial function,” “Oxidative stress,” “Age-related macular degeneration,” “Diabetic retinopathy,” and “Dry eye disease.” Inclusion criteria focused on peer-reviewed articles published in English that discussed PBM’s mechanisms at cellular and molecular levels, particularly its applications in treating ocular conditions such as age-related macular degeneration (AMD), diabetic retinopathy (DR), dry eye disease, and post-surgical recovery. Both preclinical studies, including in vitro and animal research, and clinical trials were considered. Articles were excluded if they did not focus on ophthalmic applications of PBM, were published in languages other than English, or provided insufficient or anecdotal data. Although the review did not adhere to formal systematic review protocols, care was taken to include studies of higher methodological quality, particularly those with larger sample sizes and robust experimental designs. Studies were selected based on their relevance to ophthalmic PBM, with particular attention to those demonstrating significant findings in retinal and corneal health. This approach allowed for a broad yet critical synthesis of the existing literature, providing a comprehensive picture of PBM’s potential and current applications in ophthalmology.

Mechanisms of action

**Figure 1:**
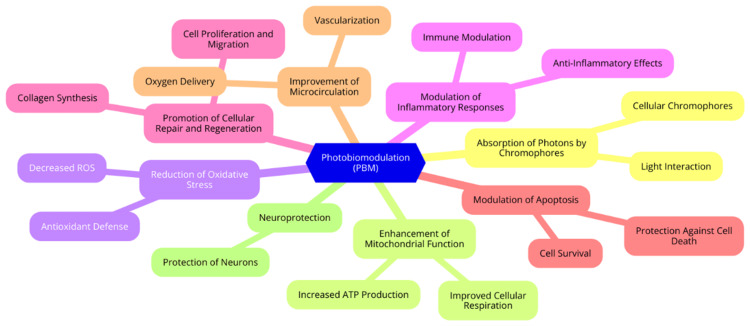
Mechanisms of action for photobiomodulation (PBM) Image Credit: Diksha Garg

The therapeutic effects of PBM are primarily attributed to its interaction with cellular chromophores, leading to both photochemical and photothermal effects. A key chromophore in this process is cytochrome c oxidase (CCO), a crucial enzyme in the mitochondrial electron transport chain [8]. When CCO absorbs low-intensity light, it triggers conformational changes that enhance the enzyme’s activity. This activation improves mitochondrial function and increases the production of ATP, which is essential for cellular energy and function. While PBM may also induce mild photothermal effects that promote vasodilation and improve blood flow, these effects are secondary to the primary photochemical interactions [9]. PBM supports retinal health through several cellular and molecular mechanisms. One of its key benefits is its ability to modulate oxidative stress and inflammation. Studies have demonstrated that PBM reduces oxidative stress by boosting the activity of antioxidant enzymes and lowering the production of reactive oxygen species (ROS) [10]. Additionally, PBM influences inflammatory pathways by decreasing the expression of pro-inflammatory cytokines and promoting the release of anti-inflammatory mediators. This dual action protects retinal cells from damage and creates an environment conducive to healing and recovery [11]. Beyond its effects on oxidative stress and inflammation, PBM enhances cellular repair and regeneration. It stimulates the expression of growth factors and promotes the proliferation and migration of vital retinal cells, such as photoreceptors and retinal pigment epithelial (RPE) cells. This enhancement of cellular repair processes is critical for restoring retinal function and preventing further degeneration. By facilitating tissue regeneration, PBM shows promise as a therapeutic intervention for various retinal conditions [12]. Lastly, PBM's impact on mitochondrial function and ATP production is central to its therapeutic efficacy. By interacting with CCO, PBM enhances mitochondrial activity and improves electron transport, increasing ATP synthesis. This boost in ATP production is crucial for maintaining cellular homeostasis and supporting the survival of retinal cells under stress. Understanding these mechanisms is essential for optimizing PBM protocols and developing more targeted, effective treatments for various retinal diseases [13]. Mechanisms of action for PBM are depicted in Figure 1.

Bench research

In Vitro Studies

In vitro studies have demonstrated that PBM can positively affect retinal and corneal cells. For retinal cells, PBM has been shown to enhance cell viability and reduce apoptosis in RPE cells and photoreceptors [14]. Research indicates that exposure to specific wavelengths (typically 600-1000 nm) improves mitochondrial function, increasing ATP production and reducing oxidative stress. Moreover, PBM has been found to modulate the release of inflammatory cytokines, a critical factor in conditions such as AMD and DR [15]. Regarding corneal cells, PBM has been studied for its effects on corneal epithelial cells, particularly in wound healing. Studies suggest that PBM promotes cell migration and proliferation, accelerating healing after corneal injuries or surgeries like LASIK. Additionally, PBM may enhance collagen synthesis and improve corneal transparency, making it a valuable tool in corneal recovery and rehabilitation [16]. The mechanisms underlying PBM’s cellular effects include mitochondrial activation, modulation of ROS, and cytokine regulation. PBM stimulates cytochrome c oxidase in the mitochondrial respiratory chain, increasing ATP production. While PBM initially elevates ROS levels, it reduces oxidative stress by enhancing antioxidant enzyme activity. Furthermore, PBM influences cytokine expression by decreasing pro-inflammatory markers and promoting anti-inflammatory responses, particularly beneficial in treating degenerative retinal diseases [17].

Animal Models

Animal models have been instrumental in elucidating the effects of PBM on retinal diseases. In studies of retinal degeneration, PBM has been shown to preserve both retinal structure and function in rodent models [18]. For example, PBM treatment has been linked to improved electroretinogram responses, reflecting enhanced retinal function and increased photoreceptor survival [18]. In diabetic animal models, PBM has effectively reduced retinal edema and inflammation, improving visual outcomes. Specifically, PBM has been found to lower the expression of vascular endothelial growth factor, a key player in the development of DR [19]. Animal studies have also explored the role of PBM in corneal wound healing. In models of corneal epithelial injury, PBM has been shown to accelerate re-epithelialization and reduce healing time by promoting keratinocyte proliferation and migration, thereby facilitating faster recovery [20]. Additionally, PBM has been investigated for its potential in post-surgical recovery, such as after corneal transplantation. These studies suggest that PBM can reduce inflammation and improve graft survival by modulating immune responses [21].

Key findings and limitations

Despite the promising findings, several gaps and challenges remain in preclinical research on PBM. A review of key results shows that PBM consistently improves cell viability in retinal and corneal cells under stress conditions. Moreover, animal studies reveal that PBM enhances functional outcomes in retinal degeneration and corneal injury models while effectively reducing inflammatory markers, which are crucial for managing retinal diseases and promoting healing [10]. However, addressing these challenges is vital for successfully transitioning PBM from laboratory research to clinical practice. One major challenge is the lack of standardized treatment protocols; variability in PBM parameters such as wavelength, intensity, and duration studies makes it difficult to compare results and establish optimal treatment regimens [22]. Furthermore, most research focuses on short-term outcomes, leaving a significant gap in understanding PBM's long-term safety and efficacy. Lastly, while animal models provide valuable insights, translating these findings to human clinical applications is complicated by differences in anatomy, physiology, and disease progression [22].

Clinical research

PBM is gaining momentum in ophthalmology, particularly its applications in treating retinal and anterior segment diseases, post-surgical recovery, and ongoing clinical trials. This innovative therapy harnesses low-intensity light to stimulate cellular processes, offering a non-invasive approach to addressing ocular conditions [5]. PBM has shown significant promise in retinal diseases, especially in managing AMD [23]. Clinical studies have reported reduced drusen volume and improved visual acuity and contrast sensitivity in patients with dry AMD [24]. For instance, research utilizing 670 nm LED light demonstrated notable enhancements in visual function among participants. PBM is also being explored as a treatment for DR, particularly diabetic macular edema (DME) [25]. Trials have shown positive outcomes, including improved visual function and reduced retinal edema after PBM treatment. One regimen involved applying LED light through a closed eyelid, effectively managing DME over extended periods. While less studied, PBM is also being investigated for its potential in other retinal conditions, such as retinal detachment, due to its ability to enhance mitochondrial function and reduce oxidative stress [25].

PBM's scope extends beyond retinal diseases to corneal and anterior segment disorders. PBM has been proposed in dry eye disease to increase tear production and reduce inflammation. Studies indicate that PBM can alleviate symptoms and relieve patients suffering from this condition [26]. Additionally, PBM has shown promise in promoting corneal wound healing, particularly for corneal ulcers, by facilitating cellular repair and potentially speeding recovery times. It is also being evaluated for its role in improving recovery after refractive surgeries like LASIK and PRK, with preliminary findings suggesting reduced inflammation and accelerated healing, leading to enhanced visual outcomes post-surgery [16]. PBM's potential in post-surgical recovery is another area of growing interest. Research suggests that PBM can reduce inflammation and promote healing after cataract surgery, with patients reporting reduced pain and faster recovery following PBM treatment. Furthermore, PBM is being investigated for its potential to enhance outcomes after conjunctival and scleral surgeries, with early findings indicating fewer post-operative complications and improved healing [27]. Regarding clinical trials, numerous studies are ongoing to assess PBM's efficacy and safety in various ophthalmic conditions. Many focus on AMD, DR, and other retinal diseases, with initial results showing promise. However, while early studies are encouraging, the evidence base is still evolving. Challenges include variability in treatment protocols and the need for larger, well-controlled trials to establish standardized guidelines. Much of the existing data comes from small trials, case series, and reports, making comprehensive meta-analyses difficult to perform [28].

Clinical integration

PBM in ophthalmology employs specific treatment parameters crucial to its efficacy [2]. The most effective PBM wavelengths typically fall within 600 nm to 1100 nm, corresponding to the optical window in human tissue. Among these, 660 nm is frequently utilized due to its ability to enhance mitochondrial activity and increase ATP production, which is particularly beneficial for retinal therapies [2]. Critical parameters for PBM include wavelength, power density, fluence, duration, and frequency. To ensure safety and effectiveness, devices used in ophthalmic PBM must meet rigorous standards, with many being FDA-approved [2]. Incorporating PBM into clinical practice requires careful attention to multiple factors. The appropriate PBM device is essential, as different devices offer varying wavelengths and power outputs. Establishing standardized treatment protocols based on current research is necessary to ensure consistent outcomes. Identifying suitable candidates, such as patients with early-stage AMD or DR, is key to maximizing the therapeutic benefits of PBM [29]. Additionally, successful implementation requires comprehensive training for ophthalmic practitioners. This includes understanding the biological mechanisms underlying PBM, technical training on device operation, and strict adherence to safety protocols [5]. Numerous case studies have demonstrated the effectiveness of PBM in treating various ocular conditions [10,23-25,30]. In one study involving 44 subjects, PBM at 670 nm improved visual acuity and reduced drusen volume, with no significant adverse effects reported [31]. Another case highlighted improvements in visual function following PBM, with patients experiencing reduced retinal edema after several treatments. Patient feedback on PBM has generally been positive, with many reporting enhanced vision and improved quality of life [32]. The non-invasive nature of PBM, combined with its minimal side effects, makes it an attractive option for patients seeking alternatives to more invasive procedures [32]. Clinical integration of PBM in ophthalmology is summarized in Table 1.

**Table 1:** Clinical integration of photobiomodulation in ophthalmology PBM: Photobiomodulation Source: [2,5,33-37]

Aspect	Description	Considerations	Current Status
Treatment Protocols	Established guidelines for PBM therapy, including parameters such as wavelength, dosage, and duration.	Customization based on specific ocular conditions and patient needs.	Protocols are evolving with ongoing research.
Device Specifications	PBM devices' characteristics include light sources (LEDs, lasers), power settings, and delivery modes.	Ensuring devices are FDA-approved and meet clinical standards.	A range of devices with varying features are available.
Training and Education	Training programs for healthcare professionals on PBM techniques, device operation, and patient management.	Need for comprehensive training to ensure effective and safe application.	Training programs are becoming more common.
Patient Selection	Criteria for selecting patients who are most likely to benefit from PBM therapy.	Assessing patient suitability based on their specific ocular conditions.	Guidelines are being refined based on clinical experience.
Outcome Assessment	Methods for evaluating the effectiveness of PBM therapy, including clinical outcomes, patient-reported outcomes, and follow-up.	Establishing standardized metrics for assessing treatment success.	Ongoing development of outcome measures and evaluation tools.
Integration with Other Therapies	Combining PBM with conventional treatments or other therapeutic modalities to enhance overall efficacy.	Coordinate with existing treatment plans and manage potential interactions.	Some evidence supports combination therapy; further research is needed.
Cost and Accessibility	Evaluation of the economic impact of PBM therapy, including cost-effectiveness and availability in different healthcare settings.	Addressing cost concerns and ensuring equitable access to therapy.	Economic evaluations are emerging; accessibility varies by region.
Regulatory Considerations	Compliance with regulatory requirements and ensuring patient safety during PBM treatments.	Adhering to regulatory guidelines and monitoring long-term safety.	Regulatory frameworks are in place, as well as ongoing updates and assessments.

Future directions and emerging trends

Ongoing research in PBM advances technology for ophthalmological applications, focusing on improving light delivery, targeting, and safety [38]. One key area of innovation involves the development of implantable light sources and fiber optic probes, which allow for direct light delivery to the retina, enhancing treatment precision. New light sources, such as superluminescent diodes, are also being investigated for their potential to increase tissue penetration depth, optimizing therapeutic outcomes. Modulating light parameters such as wavelength, pulse width, and duty cycle is another important research focus, as these adjustments can significantly impact PBM’s effectiveness while minimizing side effects [39]. While PBM has already shown efficacy in treating AMD and DR, its potential in other ophthalmic disorders is being explored through ongoing clinical trials. Conditions such as Leber's hereditary optic neuropathy, retinitis pigmentosa, and glaucoma are currently being studied to assess PBM's broader applications. Positive results from these studies could extend the use of PBM to treat retinal and optic nerve disorders with limited therapeutic options [40]. The trend is moving toward personalized treatment approaches to maximize PBM's effectiveness. Customizing PBM protocols based on disease stage, severity, and individual patient characteristics can enhance therapeutic efficacy and outcomes [41]. Moreover, integrating PBM with other treatment modalities-such as pharmacological therapies, gene therapy, or surgical techniques, produces synergistic effects, especially in complex retinal conditions. This personalized and multi-modal approach aligns with the growing trend toward precision medicine in ophthalmology, offering patients more tailored and effective care [42]. Future directions and emerging trends in PBM in ophthalmology are summarized in Table 2.

**Table 2:** Future directions and emerging trends in photobiomodulation in ophthalmology Source: [35,43-48]

Future Direction	Description	Potential Impact	Current Status
Advancements in Technology	Development of new devices with improved light sources, such as advanced LEDs and diode lasers.	Enhanced precision in treatment, broader range of applications, and improved patient outcomes.	Ongoing development with emerging technologies.
Expanded Applications	Exploration of PBM in new ophthalmic indications, such as glaucoma and retinal vein occlusion.	Potential to address a wider range of ocular conditions and improve treatment options.	Preliminary research and clinical trials.
Personalized Approaches	Tailoring PBM treatments based on individual patient characteristics and specific disease profiles.	Optimized treatment efficacy, reduced side effects, and personalized patient care.	Emerging research on personalized treatment protocols.
Combination Therapies	Integrating PBM with other therapeutic modalities, such as pharmacological treatments or regenerative medicine.	Enhanced therapeutic efficacy and potentially synergistic effects with other treatments.	Some studies on combination therapies are ongoing research.
Technological Innovations	Integration of PBM devices with modern technologies like telemonitoring and automated systems.	Improved monitoring, ease of use, and patient management.	Initial developments with ongoing advancements.
Cost-Effectiveness Analysis	Evaluating the economic impact of PBM in ophthalmic care, including cost-benefit analyses and healthcare savings.	Better understanding of PBM’s value in healthcare settings and potential cost savings.	Emerging studies and economic evaluations.
Regulatory and Safety Considerations	Addressing regulatory challenges and ensuring the long-term safety of PBM therapies.	Ensuring safe and standardised use of PBM in clinical practice.	Ongoing discussions and regulatory assessments.

## Conclusions

In conclusion, PBM represents a transformative advancement in ophthalmology, offering a range of therapeutic benefits from cellular repair to improved clinical outcomes. The review has elucidated the underlying mechanisms of PBM, demonstrating its capacity to enhance mitochondrial function, reduce oxidative stress, and promote tissue healing. A comprehensive examination of bench-to-bedside research shows that PBM holds significant promise for treating various ocular conditions, including retinal diseases, corneal disorders, and post-surgical recovery. Integrating PBM into clinical practice reflects its potential to complement existing therapies, offering a non-invasive, adjunctive approach that aligns with contemporary trends toward personalized medicine. Continuing research and clinical trials will be crucial in optimizing PBM protocols, expanding its applications, and addressing any remaining uncertainties. As the field evolves, PBM could play an increasingly pivotal role in enhancing patient care and outcomes in ophthalmology, underscoring the importance of ongoing exploration and innovation in this dynamic area of medical practice.
